# Development of a transdiagnostic digital interactive application for eating disorders: psychometric properties, satisfaction, and perceptions on implementation in clinical practice

**DOI:** 10.1186/s40337-023-00871-3

**Published:** 2023-08-29

**Authors:** Linda Booij, Mimi Israël, Manuela Ferrari, Annie St-Hilaire, Chloé Paquin-Hodge, Melissa Allard, Amélie Blaquière, Julia Dornik, Shiri Freiwald, Shawna A. Long, Marika Monarque, William D. Pelletier, Lea Thaler, Miriam Yaffe, Howard Steiger

**Affiliations:** 1 Eating Disorders Continuum, Douglas Mental Health University Institute, Montreal West Island Integrated University Health and Social Service Centre, 6603-05 LaSalle Blvd, Montreal, QC H4H 1R3 Canada; 2 Department of Psychiatry, McGill University, Montreal, Canada; 3 Research Centre, Douglas Mental Health University Institute, Montreal, Canada

**Keywords:** Digital interventions, Remote self-help, Eating disorders, Transdiagnostic, Webtool, Study implementation, Validation study

## Abstract

**Background:**

Given limited availability of informed treatments for people affected by eating disorders (EDs), there has been increasing interest in developing self-administered, technology-based ED interventions. However, many available interventions are limited to a specific ED diagnosis or assume that participants are ready to change. We developed a digital self-help application (called ASTrA) that was explicitly designed to be *transdiagnostic* and to help increase motivation for change. The aim of the present study was to describe the development and examine the psychometric properties, user satisfaction and rated potentials for practical use of our application.

**Methods:**

The content of our application was based on concepts derived from self-determination theory, the transtheoretical model of change, and cognitive theory. The application was developed by a multidisciplinary team of clinicians, researchers, staff members and individuals with lived ED experience, each being involved in all steps of the application’s development. We tested validity, reliability, satisfaction and perceived feasibility for clinical implementation in an independent sample of 15 patients with an ED and 13 clinicians specialized in ED treatment. Psychometric properties were evaluated using descriptive statistics, correlations, content validity indices and intraclass coefficients. Differences in satisfaction ratings and perceived potential for clinical implementation of the application between clinicians and patients were examined using Mann–Whitney U tests.

**Results:**

The digital application showed excellent validity (mean i-CVI: .93, range: .86–.96) and internal reliability (all Cronbach alpha’s > .88). Patients and clinicians both considered the application acceptable, appropriate, and feasible for use in clinical practice.

**Conclusions:**

Findings suggest that our transdiagnostic interactive application has excellent psychometric properties. Furthermore, patients and clinicians alike were positive about the possible use of the application in clinical practice. The next step will be to investigate the application's effectiveness as an intervention to promote autonomous motivation and to facilitate remission in people on the waitlist for specialized ED treatment.

## Abstract (el)

Several digital self-help applications have been developed to help people overcome their eating disorders. Many available interventions are limited to a specific type of eating disorder or assume that participants are ready to change. We developed a new digital self-help application (called ASTrA) that was designed for different types of eating disorders to help increase people’s motivation to recover. The aim of the present study was to describe the development and to study the psychometric properties, user satisfaction and potentials for practical use of our application. The application was developed by a group of clinicians, researchers, experts in digital health, and people who had an eating disorder in the past. We then tested validity, reliability, satisfaction and perceived feasibility for clinical implementation in an independent sample of people receiving treatment for their eating disorder and of clinicians specialized in eating-disorder treatment. We found that the application has excellent psychometric properties. Patients and clinicians alike were satisfied with the application and found the application feasible for use in clinical practice. Our next step is to study the effect of the application on symptoms and motivation in people currently on the waitlist for specialized eating-disorder treatment.

## Background

Various psychotherapeutic treatments are established as evidence-based “best practices” for the individual treatment of people with eating disorders (EDs) [1]. Foremost among these are cognitive-behavioural therapy (CBT) for bulimia nervosa (BN) [2], and enhanced CBT (CBT-E) for BN with comorbid psychopathology and anorexia nervosa (AN) [3, 4]. After these comes dialectical behaviour therapy (DBT) for BN, BN with borderline personality disorder, binge-eating disorder (BED), and AN [5, 6], suitable when there are problems of impulse- or affect regulation. The preceding treatments have a known probability of achieving positive short-term responses in many individuals with EDs [1, 7, 8].

Many people with an eating disorder respond well to the evidence-based interventions described above. However, outcome remains limited and variable, with available data indicating that only about half of people will achieve symptom abstinence by the end of a manualized, time-limited treatment regimen (For full reviews see [9–14]. Limited access to informed treatment adds a further complication—as most people affected by an ED never obtain a recommended, evidence-based treatment or, if they do, it will generally be after an inordinately long wait [14, 15].

One proposed solution to the access problem has been the development and implementation of interactive self-help applications requiring minimal guidance from health-care professionals, that can be made accessible via internet-based or other digital platforms. Aside from practical advantages related to access, self-help interventions have been thought to also help improve people’s preparation and motivation for change, initiate symptom reduction or, at least, prevent symptom progression. In line with the preceding, several available studies have tested the effectiveness of digital self-help interventions for eating disorders [16–21]. Although studies differ substantially as to design and methodology, there is accumulating evidence to suggest that self-help applications can be effective–especially for individuals with milder, or less-entrenched ED variants.

Whereas various studies have evaluated ED self-help applications in community samples, relatively few have evaluated the feasibility and effectiveness of digital interventions in individuals when offered as an adjunct to treatment-as-usual. Mobile apps involving food records or thought-monitoring (like Recovery Record) have been used to help patients practice CBT techniques between therapy sessions. However, the empirical evidence for added benefits of such practices is mixed [20]. One research group has developed and tested a video imagery-based intervention for AN that includes cognitive, motivational and behavioural components to address symptoms (vodcast) [22]. The intervention was shown to be acceptable, to enhance motivation, and to reduce ED symptoms after 3–6 weeks of regular use [23–25]. The same research group developed another digital intervention for people with AN (called RecoveryMANTRA). The intervention consists of a 6-week online guided intervention, including online self-help materials combined with weekly one-hour, individual, text-based chat sessions with a mentor (graduate psychology students) and people with lived experiences, focusing on motivation and confidence to change [26, 27]. Findings have indicated that adding RecoveryMANTRA to treatment-as-usual increased patient confidence to recover and therapeutic alliance [26, 27].

To summarize, studies have thus far shown some benefits of digital interventions for treatment of EDs, with a small number of studies focusing on digital interventions used in specialized ED treatment settings. However, we are unaware of any digital intervention that are applicable in specialized ED treatment settings, fully self-guided, transdiagnostic, and that focused explicitly on building motivation for change. The latter was of particular interest, given a demonstrated, positive relationship between pre-treatment motivation for change and later outcome (see [28–30]).

This paper describes the development and psychometric properties of the application in question, called “Auto-Soins pour les Troubles de l’Alimentation” (ASTrA) (French for “self-help for eating disorders”). We examined face validity, content validity, and reliability. We also examined patients' and clinicians’ ratings of the application’s acceptability, and the extent to which they perceived the application to be suitable for use in clinical practice.

## Methods

### Development of the digital application ASTrA

We established a working group consisting of clinicians working in our specialized program for ED treatment (Eating Disorders Continuum, Douglas Mental Health University Institute), collaborating researchers affiliated with the Research Centre of the Douglas Mental Health University Institute, and a group of people with lived experience with an ED. Most of the clinicians involved also had experience in research on EDs. The working group was led by a senior clinician-researcher with more than 35 years of experience in clinical and research activity in the EDs (HS) and included a clinician-researcher and research methodologist with extensive research expertise in EDs (LB), four clinician-researchers from the EDC team, three team clinicians, a psychiatry fellow, a researcher with expertise in the development of digital interventions for mental disorders, one support staff member working in a digital health studio, and three individuals with lived experiences. Following a review of the scientific literature and first discussions by HS with international colleagues who had experience with developing digital eating-disorder intervention applications, members of the working group met regularly (1–2 times per month between August 31 2021 and June 13 2022), to discuss the development of the application. The group subsequently reached consensus about the specific modality of the application (i.e., an application with interactive exercises), relevant theoretical frameworks (self-determination theory, transtheoretical model of change and cognitive-behavioural model of eating disorders), specific eating-disorder symptom dimensions to assess (preoccupation with shape and weight), and response format (combination of multiple-choice and constructed-response format) of the application. Clinician members of the team subsequently drafted scripts for the interactive exercises (“items”), addressing common beliefs about eating, body image and weight control. HS further reviewed and edited the team members’ contributions, in collaboration with other members of the team. After a group voting process and further discussion, twelve scenarios were retained, which were in turn further revised by team members (both clinicians and individuals with lived experiences) for clarity, familiarity, and fidelity with the intended theoretical construct. Bilingual members of the team then translated each of the scenarios, originally written in English, to French and then back again to English, using a back-translation procedure. Next, the instructions and interactive items of the ASTrA application were programmed. The working group agreed on the final design and look of the application and performed usability testing to debug the digital tool.

The application was centered around four core clinical features relevant for most types of eating disorders: (i) fear of gaining weight (ii) obsessions around weight, shape and eating, (iii) ambivalence around change and (iv) binge-eating. Addressing the preceding core symptoms is in line with evidence-informed approaches in the treatment of different types of eating disorders [31, 32].

Each of the 12 items of the ASTrA application started with a description of a maladaptive eating-disorder-related belief/cognition, followed by one or more questions designed to promote reflection and re-evaluation of the belief (called “feedback for reflection”). For most of the items, the reflection question was followed by either a follow-up reflection question or a text providing additional psychoeducational information. If the participant’s answer to the reflection question suggested the presence of a maladaptive cognition around shape, weight or eating, the follow-up reflection question was designed to further challenge the maladaptive cognition. If the participant’s answer to the reflection question indicated a healthy cognition around shape, weight or eating, a confirmative response appeared on the screen (e.g., “indeed”, “good thinking”), followed by a brief psychoeducational description of why the chosen answer was correct. Next, the following item appeared on the screen until all 12 items were addressed. Participants could go back at any time to any of the content of an item by pressing a backward navigation button on the screen. The ASTrA application was programmed in a way that participants were not able to fully skip any item. See Table 1 for a summary of each of the scenarios.

**Table 1:** Themes of the items of the ASTrA application and a description of the content

#	Theme of the item	Description of the content of the item
1	Do I have a weight problem, or more a fear of gaining weight?	*Common thinking patterns:* When I eat, I get anxious, panic and hate myself for letting myself go. I am very afraid of gaining weight *Feedback for reflection:* Is it your weight that’s the source of your unhappiness? Or is it the constant fear of gaining weight? *Choice:* Weight/Fear Depending on the participant’s answer, more detailed reflective follow-up questions appear on the screen about the possible cause of distress (weight *vs*. fear of gaining weight). The idea of EDs as a weight gain phobia is explained and how by gradually letting go of restricting, a person can discover that there is no need to restrict to control weight
2	Does losing weight solve my problem?	*Common thinking patterns:* If only I can get my weight down a bit more, I’ll feel better. More confident. More attractive. More in control *Feedback for reflection:* Does losing weight really help you feel better? *Choice:* Yes/No Depending on the participant’s answer, reflective follow-up questions appear on the screen about whether losing weight causes temporary positive feelings such as more self-confidence *vs*. whether losing weight did not make fears of gaining weight or body-image concerns go away in the long term
3	Set point is nature’s parachute	*Common thinking patterns:* I feel that I must be very careful about how much I eat. When I eat even a little more, my weight goes up. I see it on the scale right away. It’s like I gain weight very easily. Is it really supposed to take so much work to avoid weight gain? *Feedback for reflection:* Are you trying to maintain your weight below the one that is natural for you? *Choice:* Yes/No, followed by psychoeducational information about set-point. Specific content depends on the answer of the participant
4	You cannot maintain your weight (unless at your set point)	*Common thinking patterns:* My weight is pretty good now…and it’s as high as I want it to go…any higher and I’ll want to restrict some more. After all, I’m not physically ill anymore, I’m not in any danger. I’ll just watch what I eat and keep the weight I have now *Feedback for reflection:* Do you think it is possible to maintain your weight below your Set Point? *Choice:* I can try/Don’t even try Depending on the participant’s answer, reflective questions appear on the screen around the feasibility to maintain a weight below set point *vs*. how a weight below set point could re-trigger a cycle of being over-cautious and undereating
5	No weight is good enough	*Common thinking patterns:* If only I was able to get down to “xx” kilograms, I would feel better, more confident and I could stop worrying about my weight all the time. Just a little more ought to do it *Feedback for reflection:* Really? Have you ever achieved a weight at which discomfort and worry about your weight and body image really went away, and no longer left you distressed? *Choice:* Yes/No Depending on the participant’s answer, reflective questions appear on the screen around whether weight loss leads to positive feelings that are temporary *vs*. whether weight loss made body preoccupations and worries about the risk of regaining weight not go away
6	Paradoxically, you reduce binge-eating by eating more	Common thinking patterns: I have to be careful not to eat past a certain point, because if I do it will trigger a binge. I don’t know what’s with me. I just can’t control myself. I’m some kind of food junkie *Feedback for reflection*: Are you someone who can’t say “No” to food? Or is it more likely that you are chronically under-fed, or very often thinking about how you need to eat less? For example, when was the last time you regularly ate 3 meals a day? *Choice:* It’s been a long time/ I always eat 3 balanced meals/day Depending on the participant’s answer, reflective questions appear on the screen around whether restriction or thinking about dieting/restricting could increase risk for bingeing *vs*. how regular eating helps to control appetite
7	Full is not fat	*Common thinking patterns:* I hate feeling full. I can see my stomach bloat and I feel fat *Feedback for reflection:* People with eating disorders often complain that they are prone to uncomfortable feelings of fullness or bloating, or uncomfortable stomach acidity, burning, reflux, or cramps. They even say that their stomachs bloat out after eating relatively small amounts of food. Do you have that? Do you stop eating before you feel full in order to avoid the uncomfortable feeling of fullness? The preceding reflective questions are followed by presenting information about how regular eating reduces bloating or other digestive issues
8	Rapid weight gain (or loss) is not real	*Common thinking patterns:* I am someone who gains weight very easily, so I have to be very cautious about what or how much I eat *Feedback for reflection:* Human bodies don’t gain or lose body mass very rapidly. Body mass change is a very slow process that takes weeks, or even months. Rapid weight changes are due to changes in hydration (water). You lose water (by sweating, urinating, restricting, or purging). Your body can gain (or lose) several kilos of water weight very quickly (within a day). Many people get fooled into reacting to (or compensating for) meaningless, rapid changes in weight that are due only to water retention and water loss The rule is: If the change happened quickly, it is NOT REAL
9	Are you the person you want to be?	*Point for reflection:* When acting on your eating disorder (dieting, restricting, controlling weight, being body preoccupied, etc.) are you really being true to your own values? I mean are you giving priority to the things that really matter to you? To help you consider this question, please click the values that you consider to be the most important for you. *[table with 62 values, values light up as they are clicked]* Are you giving more space to the values that are important to your eating disorder or to those that are important to you? Can you make more space for your own values? YOUR priorities? Taking time to identify your values helps to give meaning to your life, especially if you behave in a way that is aligned with your values. Can you think of specific actions you could do this week that would bring you closer to your values?
10	There are other ways to manage emotions besides relying on eating-disorder behaviours	*Common thinking patterns:* When my emotions get really strong, I can’t handle them. They overwhelm me. My eating disorder helps me escape from strong emotions, or at least to keep them in check. *Feedback for reflection:* When you try to avoid, push away, or escape from emotions, it may work in the short-term. But, have you noticed that the emotions tend to come back, sometimes even stronger? Eating disorder symptoms can be a coping mechanism people develop to push away their emotions. This can “work” in the short-term but tends to bring about various negative consequences. Using the eating disorder to deal with emotions can block you from developing more-effective ways of coping with emotions and comes at a high price. Which of the following are part of the price you pay for your eating disorder symptoms? [*14 problem behaviours appear*] One of the things we work on in eating-disorder treatment is developing effective ways of soothing emotions
11	Being thinner is the key to happiness	*Common thinking patterns:* People tell me to stop dieting and to stop watching my weight. They say that I don’t eat enough. But I like what I see in the mirror right now. Why should I change that? I don’t tell them what to do *Point for reflection:* Even though weighing less has some positive aspects for you, are you sure it’s worth it? *Choice:* Yes/No Depending on the participant’s answer, reflective questions on whether or not weighing less is worth it in the long run. The participant is then asked to indicate what percentage of mental energy goes into thinking about weight, body image and eating (< 30%, 30–50%, 50–75%, 75–100%), and asked to reflect on whether or not that percentage is too much (choice: yes/no). If answer is no, a checklist of six possible psychological costs of eating, weight and body-image concerns appears, and the participant is asked to reflect and tick all that apply
12	I need to “make room” to let myself eat	*Common thinking patterns:* In order to allow myself to eat, I must first burn the calories either by exercising, purging or by restricting my intake. The higher the energy expenditure, the more I am allowed to eat *Feedback for reflection:* Did you know that your body needs energy even when you are at rest? The body needs fuel (and therefore food) to function properly, even if you haven’t exercised. It isn’t necessary to use compensating behaviors to give yourself permission to eat. Your body actually requires energy to function! Psychoeducation is given around the body’s energy needs

### Participants

Following development and programming, the digital self-help application ASTrA was assessed quantitatively in an independent sample consisting of adult patients who were receiving treatment at the Eating Disorders Continuum and clinicians (permanent staff or graduate-level trained clinical interns) working in our specialized eating-disorder program.

All patient participants were diagnosed with AN, BN or other specified feeding or eating disorder (OSFED) as defined by DSM-5 criteria. Diagnosis was determined by experienced clinicians following semi-structured interviews and confirmed by multidisciplinary team consensus. Since the number of individuals offered treatment services in our specialized program with BED is rather small, our sample did not include individuals with BED. We also excluded people with avoidant/restrictive food intake disorder (ARFID), as our application was not designed for individuals with this diagnosis.

All participants provided written informed consent prior to participation. The study was approved by the Research Ethics Board of the Douglas Mental Health University Institute (REB: 2023-633).

### Measures

#### Technical stability

Each participant was asked to test out certain technical features of ASTrA such as back and forward navigation, clickable fields, loading of webpages, and was invited to provide comments on each of the preceding technical features.

#### Face and content validity

The items to assess the validity of ASTrA were constructed using guidelines of the consensus-based standards for the selection of health measurement instruments (COSMIN) for evaluating content validity [33]. Participants were asked to rate on a 4-point scale for each item comprehensibility (3 items, including clarity, easy to understand and free of technical language), presence/absence of objective errors, relevance, as well as presence/absence of typos. Each item consisted of a 4-point scale, where 1 = strongly disagree, 2 = disagree, 3 = agree, and 4 = strongly agree.

#### Acceptability, appropriateness, and perceived feasibility

Participants completed the Acceptability of Intervention Measure (AIM), Intervention Appropriateness Measure (IAM), and the Feasibility of Intervention Measure (FIM) [34, 35]. The AIM, IAM and FIM scales each consist of 4 items, rated on a 5-point scale (1 = completely disagree, 2 = disagree, 3 = neither agree nor disagree, 4 = agree, 5 = completely agree), designed to assess the acceptability, appropriateness, and feasibility of an intervention. The AIM, IAM and FIM measures are based on the theoretical framework by Procter et al. (2011) [36], postulating that clinicians’ perceived acceptability, appropriateness, and feasibility of a new intervention are key indicators of actual implementation success. The AIM, IAM and FIM developed by Weiner et al. (2017) showed good–excellent psychometric properties [35].

### Statistics

Descriptive statistics were used to examine mean ratings on the outcome measures. Mann–Whitney *U* tests (two-tailed) were used to test for differences in mean ratings of each of the outcome measures between patients and clinicians or between subgroups of patients. To quantify the content validity of the individual items of the digital application, the item-level content validity index (i-CVI) was calculated, using the relevance rating for each of the 12 items of the application, measured on a 4-point scale. i-CVI was computed by measuring the number of times participants rated the relevance of a particular item as a “3” (agree) or “4” (strongly agree), divided by the total number of participants who rated the item [37]. According to the criteria of Polit et al. (2007) [37], in the case of three of more raters, an i-CVI larger than 0.78 is considered excellent [37]. Content validity for the overall application (sum of the content validity index, s-CVI) was computed by taking the average i-CVI scores across the 12 individual items of the application [37].

To examine the reliability, Cronbach’s alpha coefficients were calculated to assess the level of consistency across the 12 items in terms of comprehensibility, absence of objective errors and relevance of the ASTrA application. A Cronbach’s alpha higher than 0.80 is typically indicative of good internal consistency [38].

### Power analysis

There is no consensus about the number of raters required to perform a content validity evaluation [33, 39, 40]. Although the specific number of raters depends on the required expertise and the range of knowledge among evaluators, a sample size between 3–10 for each group of experts (professionals and lay experts) is considered acceptable [39].

To support Cronbach’s alpha coefficients, we based ourselves on the formula of Bonett et al. [41] and followed the recommendations of Bujang et al. [42]. Specifically, with the null hypothesis of a coefficient of Cronbach’s alpha set at 0.50 and the alternative hypothesis of a Cronbach’s alpha set at 0.80, the minimum sample size to determine the internal consistency of the ratings for the 12 items is 22 participants, with a power of 0.80. We therefore considered the present sample (15 individuals with eating disorders and 13 clinicians working with individuals with eating disorders) sufficient to examine the research questions of the present study.

## Results

### Participants

Fifteen patients and 14 clinicians (9 professionals, 5 graduate trainees) were recruited for this study. One participant (a health professional) did not complete 3 items and his/her data were therefore excluded from analyses. Two other participants (one health professional, one patient) missed one item and were retained in the analyses, resulting in a final sample of 15 patients and 13 clinicians. Patients were diagnosed with AN-Restrictive type (AN-R, n = 6), AN-Binge-eating/purging type (AN-BP, n = 6), BN (n = 2), or OSFED-purging disorder (n = 1). Clinicians had a background in psychology (n = 5), social work (n = 3), psycho-education (n = 3) or medicine/nursing (n = 2), with an average experience of 6.38 years (SD = 10.9 years, range 0–38 years) in working with people with eating disorders. All participants were females.

#### Technical stability

All individuals indicated that the ASTrA application was easy to navigate, and that all fields and web pages loaded properly.

#### Face and content validity

Participants agreed/strongly agreed that the items were comprehensible (clear, easy to understand, free of technical knowledge), free of objective errors, and relevant (Table 2). Some suggestions were also given to improve the grammar. There were overall no differences in any of the item ratings between clinicians and patients, except that patients found item 2 (“does losing weight solve my problem?”) easier to understand than clinicians (Z = − 2.03, p = 0.04). There were also no significant differences in ratings between patients with a restrictive spectrum ED (AN-R, n = 6) and patients who presented with a bulimic spectrum ED (AN-BP, BN, or OSFED, purging disorder, n = 9).

**Table 2:** Descriptive statistics of the items of the application

#	Theme of the item	Clarity	Understandability	Free of technical language	Free of objective errors	Relevance	i-CVI
1	Do I have a weight problem, or more a fear of gaining weight?	3.39 ± 0.7	3.39 ± 0.8	3.57 ± 0.7	3.61 ± 0.6	3.75 ± 0.5	.96
2	Does losing weight solve my problem?	3.36 ± 0.8	3.61 ± 0.7	3.75 ± 0.6	3.57 ± 0.7	3.64 ± 0.7	.86
3	Set point is nature’s parachute	3.25 ± 1.0	3.43 ± 0.8	3.46 ± 0.8	3.36 ± 0.8	3.64 ± 0.6	.93
4	You cannot maintain your weight (unless at your set point)	3.00 ± 0.9	3.29 ± 0.8	3.64 ± 0.5	3.25 ± 0.8	3.61 ± 0.6	.93
5	No weight is good enough	3.46 ± 0.8	3.57 ± 0.7	3.71 ± 0.5	3.46 ± 0.7	3.71 ± 0.5	.96
6	Paradoxically, you reduce binge-eating by eating more	3.57 ± 0.6	3.50 ± 0.7	3.64 ± 0.6	3.50 ± 0.7	3.71 ± 0.6	.93
7	Full is not fat	3.68 ± 0.6	3.71 ± 0.6	3.71 ± 0.5	3.61 ± 0.7	3.79 ± 0.6	.93
8	Rapid weight gain (or loss) is not real	3.52 ± 0.7	3.63 ± 0.6	3.74 ± 0.5	3.52 ± 0.7	3.81 ± 0.6	.93
9	Are you the person you want to be?	3.54 ± 0.8	3.46 ± 0.8	3.68 ± 0.6	3.36 ± 0.8	3.75 ± 0.6	.93
10	There are other ways to manage emotions besides relying on eating-disorder behaviours	3.75 ± 0.5	3.78 ± 0.5	3.74 ± 0.5	3.56 ± 0.6	3.74 ± 0.6	.93
11	Being thinner is the key to happiness	3.74 ± 0.5	3.78 ± 0.4	3.78 ± 0.4	3.48 ± 0.8	3.81 ± 0.5	.96
12	I need to “make room” to let myself eat	3.54 ± 0.8	3.61 ± 0.7	3.79 ± 0.4	3.50 ± 0.7	3.71 ± 0.5	.96
1–12	Mean scores ± SD across 12 items	3.48 ± 0.5	3.56 ± 0.5	3.68 ± 0.4	3.48 ± 0.6	3.72 ± 0.5	.93

(Mean ± *SD*) for each of the items of ASTrA application, rated on a scale with 1 = strongly disagree, 2 = disagree, 3 = agree, 4 = strongly agree, as well as the item-level Content Validity Index (i-CVI) for the relevance items of the digital application

Item-CVI for each of the 12 items ranged from 0.86 to 0.96 (see Table 2), whereas the s-CVI was 0.93, indicating excellent content validity. Content validity was also considered excellent when analyzing the CVI indices separately for patients and clinicians (i-CVI range for patients: 0.80–0.93, s-CVI = 0.88; range i-CVI clinicians: 0.92–1, s-CVI: 0.99).

*Reliability.* The overall Cronbach’s alpha across all ratings of the 12 items was excellent (alpha = 0.97, 95% CI 0.95–0.98). The internal consistency was also considered very good to excellent for each of the individual rating scales, including clarity (alpha = 0.88, 95% CI 0.80–0.94), understandability (alpha = 0.89, 95% CI 0.82–0.94), free of technical language (alpha = 0.93, 95% CI 0.89–0.97), absence of objective errors (alpha = 0.94, 95% CI 0.90–0.97), and relevance (alpha = 0.96, 95% CI 0.93–0.98).

#### Acceptability, appropriateness, and perceived feasibility

Cronbach’s alpha across the 12 AIM, IAM and FIM items in the present study was excellent (alpha = 0.93).

**Fig. 1:**
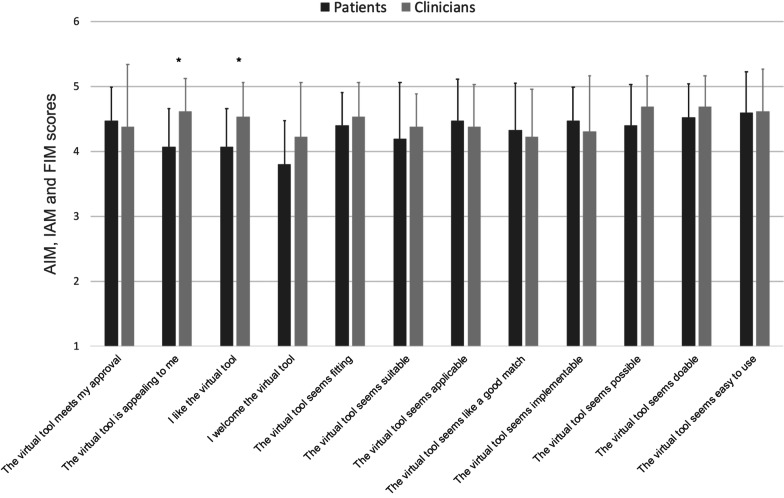
Acceptance, appropriateness and feasibility ratings of ASTrA, from a patient and clinician perspective. Ratings vary from 1 = completely disagree to 5 = completely agree. Values represent Means ± SD. *p < .05 (2-tailed). AIM = Acceptability of Intervention Measure; IAM = Intervention Appropriateness Measure; FIM = Feasibility of Intervention Measure

Both patients and clinicians agreed – completely agreed that the ASTrA application (mean ± *SD*) was acceptable (4.26 ± 0.5), appropriate (4.37 ± 0.6) and feasible (4.54 ± 0.5). There were no differences in ratings between patients and clinicians on AIM, IAM and FIM total scores (*p* > 0.14). There were also no differences as to the acceptability, appropriateness, and perceived feasibility between patients with a restrictive spectrum ED (AN-R, n = 6) and patients who presented with a bulimic spectrum ED (AN-BP, BN, or OSFED, purging disorder, n = 9). At the individual item level, clinicians rated ASTrA as slightly more appealing than patients (Z = − 2.36, *p* = 0.02) and liked ASTrA slightly more than patients (*Z* = − 2.05, *p* = 0.04). Figure 1 displays the means (*SD*) of each of the individual AIM, IAM and FIM questions, stratified by group.

## Discussion

This paper describes the development of a digital self-help application (ASTrA) designed to promote re-evaluation of beliefs associated with ED symptoms and to enhance motivation for change, and to test the psychometric properties of the application and examine patients' and clinicians’ satisfaction with the application and their ratings of its suitability for implementation in clinical practice.

The ASTrA application had excellent psychometric properties; internal reliability was high, and the application showed excellent face and content validity. Furthermore, using standardized questionnaires on knowledge implementation feasibility, patients and clinicians were satisfied with the application and perceived it as relevant, feasible and acceptable to implement in clinical practice.

Over the years, numerous digital interventions for the treatment of eating disorders have been developed, and many of them have shown effectiveness in reducing eating-disorder symptomatology [16–21]. However, most applications are designed for people with a specific ED diagnosis (e.g., only AN or only BN), or assume participants' readiness to change. The few digital eating-disorder applications that include motivational components commonly require guidance from a therapist or peer mentor, or are designed solely for people with AN. To our knowledge, our ASTrA application is a first that is fully self-guided, includes both motivational and cognitive components and is suitable for different types of eating disorders. The use of such application in the context of specialized eating-disorder treatment is relevant at the present times, considering the increased number of people on the waitlist for eating-disorder treatment (e.g. [43, 44], and the shortage of mental health professionals [45].

The present study has some limitations: Whereas the content of ASTrA is relevant for any eating disorder in which shape and weight concerns are central (i.e., AN, BN, and certain forms of OSFED), most items are not relevant for ARFID. It is also important to mention that the patient sample in the present study consisted primarily of people diagnosed with AN. Although there were no significant differences in ratings between patients with a bulimic-spectrum eating-disorder presentation (AN-BP, BN, purging disorder) and patients with a restrictive-spectrum presentation (AN-R), few participants had a diagnosis of BN or OSFED. The preceding means that future studies examining the relevance and psychometric properties of the ASTrA application should include samples implicating more varied ED diagnoses, so as to ensure generalizability. Additionally, whereas the present study only described users’ experiences with the application, further research is needed to evaluate the application’s clinical effectiveness. Such work is currently underway in our eating-disorder program. Finally, the psychometric study was conducted with a non-random sample. As in most studies on digital applications, the preceding may imply that our study may have been subject to selection bias, which could have impacted the ratings. We aimed to minimize bias by explicitly stating in the instructions that we were interested in their perception and appreciation of the interactive digital application. In addition, each item was followed by an invitation to provide constructive comments, such as flagging sentences and text that are unclear or potentially missing portions of the content, and to report any other issues.

Strengths of the study are that the ASTrA application was based on key theoretical motivational frameworks relevant to eating disorders, designed according to best practices of test construction, and a result of a collaborative effort between clinicians, individuals with lived experiences and researchers, each being involved in all steps of the application’s development.

Our next step is to conduct a randomized controlled trial testing the effectiveness of ASTrA to enhance motivation and reduce symptom severity among individuals waitlisted for specialized ED treatment. If effective, implementing such application in clinical practice would be an essential step forward in improving access to and optimizing specialized eating-disorder care.

## Conclusion

Findings suggest that our transdiagnostic interactive digital application ASTrA has excellent psychometric properties. Additionally, patients and clinicians were positive about the use of the application in clinical practice. The next step will be to investigate the application's effectiveness as an intervention among people on the waitlist for specialized ED treatment.

## Data Availability

The datasets generated and/or analysed during the current study are not publicly available due to privacy restrictions but part of the data may be available from the corresponding authors on reasonable request.

## Abbreviations

AIM: Acceptability of Intervention Measure
AN: Anorexia nervosa
ARFID: Avoidant/restrictive food intake disorder
ASTrA: Auto-Soins pour les Troubles de l’Alimentation (french for “self-help for eating disorders”)
BED: Binge-eating disorder
BN: Bulimia nervosa
CBT: Cognitive-behavioural therapy
COSMIN: Consensus-based standards for the selection of health measurement instruments
DBT: Dialectical behaviour therapy
DSM-5: Diagnostic and statistical manual of mental disorders, fifth edition
EDs: Eating disorders
FIM: Feasibility of Intervention Measure
IAM: Intervention Appropriateness Measure
i-CVI: Item-level, content validity index
s-CVI: Sum of the content validity index
SD: Standard deviation
OSFED: Other Specified Feeding and Eating Disorders

## References

[1] Treasure J, Duarte TA, Schmidt U (2020). Eating disorders. Lancet.

[2] Fairburn CG, Marcus MD, Wilson GT, Fairburn CG, Wilson GT (1993). Cognitive-behavioral therapy for binge eating and bulimia nervosa: A comprehensive treatment manual. Binge eating: Nature, assessment and treatment New York.

[3] Fairburn CG, Cooper Z, Doll HA, O'Connor ME, Bohn K, Hawker DM (2009). Transdiagnostic cognitive-behavioral therapy for patients with eating disorders: a two-site trial with 60-week follow-up. Am J Psychiatry.

[4] Frostad S, Calugi S, Engen CBN, Dalle GR (2021). Enhanced cognitive behaviour therapy (CBT-E) for severe and extreme anorexia nervosa in an outpatient eating disorder unit at a public hospital: a quality-assessment study. J Eat Disord.

[5] Federici A, Wisniewski L (2013). An intensive DBT program for patients with multidiagnostic eating disorder presentations: a case series analysis. Int J Eat Disord.

[6] Rozakou-Soumalia N, Darvariu S, Sjogren JM. Dialectical behaviour therapy improves emotion dysregulation mainly in binge eating disorder and bulimia nervosa: a systematic review and meta-analysis. J Pers Med. 2021;11(9). 10.3390/jpm11090931 PMC8470932 34575707

[7] Monteleone AM, Pellegrino F, Croatto G, Carfagno M, Hilbert A, Treasure J (2022). Treatment of eating disorders: a systematic meta-review of meta-analyses and network meta-analyses. Neurosci Biobehav Rev.

[8] Ben-Porath D, Duthu F, Luo T, Gonidakis F, Compte EJ, Wisniewski L (2020). Dialectical behavioral therapy: an update and review of the existing treatment models adapted for adults with eating disorders. Eat Disord.

[9] Kaidesoja M, Cooper Z, Fordham B (2023). Cognitive behavioral therapy for eating disorders: a map of the systematic review evidence base. Int J Eat Disord.

[10] Galsworthy-Francis L, Allan S (2014). Cognitive behavioural therapy for anorexia nervosa: a systematic review. Clin Psychol Rev.

[11] Groff SE (2015). Is enhanced cognitive behavioral therapy an effective intervention in eating disorders? A review. J Evid Inf Soc Work.

[12] Hay PJ, Bacaltchuk J (2003). Psychotherapy for bulimia nervosa and binging. Cochrane Database Syst Rev.

[13] Lock J (2015). An update on evidence-based psychosocial treatments for eating disorders in children and adolescents. J Clin Child Adolesc Psychol.

[14] Pehlivan MJ, Miskovic-Wheatley J, Le A, Maloney D, Research Consortium NED, Touyz S, et al. Models of care for eating disorders: findings from a rapid review. J Eat Disord. 2022;10(1):166. 10.1186/s40337-022-00671-1 PMC9667640 36380363

[15] Kazdin AE, Fitzsimmons-Craft EE, Wilfley DE (2017). Addressing critical gaps in the treatment of eating disorders. Int J Eat Disord.

[16] Aardoom JJ, Dingemans AE, Spinhoven P, Van Furth EF (2013). Treating eating disorders over the internet: a systematic review and future research directions. Int J Eat Disord.

[17] Ahmadiankalati M, Steins-Loeber S, Paslakis G (2020). Review of randomized controlled trials using e-health interventions for patients with eating disorders. Front Psychiatry.

[18] Anastasiadou D, Folkvord F, Lupianez-Villanueva F (2018). A systematic review of mHealth interventions for the support of eating disorders. Eur Eat Disord Rev.

[19] Schlegl S, Burger C, Schmidt L, Herbst N, Voderholzer U (2015). The potential of technology-based psychological interventions for anorexia and bulimia nervosa: a systematic review and recommendations for future research. J Med Internet Res.

[20] Dufour R, Novack K, Picard L, Chadi N, Booij L (2022). The use of technology in the treatment of youth with eating disorders: a scoping review. J Eat Disord.

[21] Barakat S, Maguire S. Accessibility of psychological treatments for bulimia nervosa: A review of efficacy and engagement in online self-help treatments. Int J Environ Res Public Health. 2022;20(1). 10.3390/ijerph20010119 PMC9819826 36612445

[22] Treasure J, Macare C, Mentxaka IO, Harrison A (2010). The use of a vodcast to support eating and reduce anxiety in people with eating disorder: A case series. Eur Eat Disord Rev.

[23] Cardi V, Esposito M, Clarke A, Schifano S, Treasure J. The impact of induced positive mood on symptomatic behaviour in eating disorders. An experimental, AB/BA crossover design testing a multimodal presentation during a test-meal. Appetite. 2015;87:192–8. 10.1016/j.appet.2014.12.224 25555537

[24] Cardi V, Kan C, Roncero M, Harrison A, Lounes N, Tchanturia K (2012). Mealtime support in anorexia nervosa: a within-subject comparison study of a novel vodcast intervention. Psychother Psychosom.

[25] Kim YR, Cardi V, Lee GY, An S, Kim J, Kwon G (2019). Mobile self-help interventions as augmentation therapy for patients with anorexia nervosa. Telemed J E Health.

[26] Cardi V, Albano G, Ambwani S, Cao L, Crosby RD, Macdonald P (2020). A randomised clinical trial to evaluate the acceptability and efficacy of an early phase, online, guided augmentation of outpatient care for adults with anorexia nervosa. Psychol Med.

[27] Cardi V, Leppanen J, Leslie M, Esposito M, Treasure J (2019). The use of a positive mood induction video-clip to target eating behaviour in people with bulimia nervosa or binge eating disorder: an experimental study. Appetite.

[28] Sansfacon J, Gauvin L, Fletcher E, Cottier D, Rossi E, Kahan E (2018). Prognostic value of autonomous and controlled motivation in outpatient eating-disorder treatment. Int J Eat Disord.

[29] Steiger H, Sansfacon J, Thaler L, Leonard N, Cottier D, Kahan E (2017). Autonomy support and autonomous motivation in the outpatient treatment of adults with an eating disorder. Int J Eat Disord.

[30] Thaler L, Israel M, Antunes JM, Sarin S, Zuroff DC, Steiger H (2016). An examination of the role of autonomous versus controlled motivation in predicting inpatient treatment outcome for anorexia nervosa. Int J Eat Disord.

[31] Fairburn CG (2008). Cognitive-behavior therapy for eating disorders.

[32] Waller GCH, Corstorphine E, Hinrichsen H, Lawson R, Mountford V, Russell K (2007). Cognitive behavioral therapy for eating disorders: a comprehensive treatment guide.

[33] Terwee CB, Prinsen CAC, Chiarotto A, Westerman MJ, Patrick DL, Alonso J (2018). COSMIN methodology for evaluating the content validity of patient-reported outcome measures: a Delphi study. Qual Life Res.

[34] Mendelson D, Thibaudeau E, Sauve G, Lavigne KM, Bowie CR, Menon M (2022). Remote group therapies for cognitive health in schizophrenia-spectrum disorders: feasible, acceptable, engaging. Schizophr Res Cogn.

[35] Weiner BJ, Lewis CC, Stanick C, Powell BJ, Dorsey CN, Clary AS (2017). Psychometric assessment of three newly developed implementation outcome measures. Implement Sci.

[36] Proctor E, Silmere H, Raghavan R, Hovmand P, Aarons G, Bunger A (2011). Outcomes for implementation research: conceptual distinctions, measurement challenges, and research agenda. Adm Policy Ment Health.

[37] Polit DF, Beck CT, Owen SV (2007). Is the CVI an acceptable indicator of content validity? Appraisal and recommendations. Res Nurs Health.

[38] Schober P, Mascha EJ, Vetter TR (2021). Statistics from a (agreement) to z (z score): a guide to interpreting common measures of association, agreement, diagnostic accuracy, effect size, heterogeneity, and reliability in medical research. Anesth Analg.

[39] Rubio DM, Berg-Weger M, Tebb SS, Lee ES, Rauch S (2003). Objectifying content validity: conducting a content validity study in social work research. Soc Work Res.

[40] Grant JS, Davis LL (1997). Selection and use of content experts for instrument development. Res Nurs Health.

[41] Bonett DG (2002). Sample size requirements for testing and estimating coefficient alpha. J Educ Behav Stat.

[42] Bujang MA, Omar ED, Baharum NA (2018). A review on sample size determination for Cronbach's Alpha Test: a simple guide for researchers. Malays J Med Sci.

[43] Devoe D, Han A, Anderson A, Katzman DK, Patten SB, Soumbasis A (2022). The impact of the COVID-19 pandemic on eating disorders: a systematic review. Int J Eat Disord.

[44] Taquet M, Geddes JR, Luciano S, Harrison PJ. Incidence and outcomes of eating disorders during the COVID-19 pandemic. Br J Psychiatry. 2021:1–3. 10.1192/bjp.2021.105 PMC7612698 35048812

[45] Moroz N, Moroz I, D'Angelo MS (2020). Mental health services in Canada: barriers and cost-effective solutions to increase access. Healthc Manage Forum.

